# Action on the Death of William Warren Potter

**Published:** 1911-05

**Authors:** 


### Action on the Death of William Warren Potter

At a meeting of the Alumni Association of the Buffalo General Hospital, held April 17, 1911, the following memorial was adopted and ordered spread upon the records, and also that a copy be sent to the BUFFALO MEDICAL JOURNAL.

### IN MEMORIAM.

Forasmuch as it hath pleased Almighty God in his wise providence to take out of this world the soul of our fellow member William Warren Potter, for many years Consulting Surgeon to the Buffalo General Hospital, we, therefore, recall with grateful appreciation the significant fact, that previous to his appointment upon this staff, Dr. Potter had won and adorned the highest official positions in the gift of the members of our profession in the County and in the State, as well as the highest responsibility and honor in the gift of the Regents of the University of the State of New York, to-wit: the presidency of the Medical Society of the County of Erie (1893), the presidency of the Medical Society of the State of New York (1891), and the presidency of the State Board of Medical Examiners (1897). Ripe with these honors, measured, tried and tested with these preferments, Dr. Potter paid an emphatic compliment to this staff in accepting its offer of membership. It is, therefore, meet and fit that our official records should bear evidence of the high appreciation of the entire medical profession for the unusual qualities of mind, character and personality with which Dr. Potter distinguished that profession, and by which he won his many and singularly exalted positions.

It may also be added that Dr. Potter bore the severe test of character, a clear intimation that his days were numbered and that death might not be long delayed, was near at hand, with that calm serenity, that cheerful resignation and preparedness becoming the poise and courage of the physician, philosopher and soldier, confident of victory in the greatest of life's conflicts.

May light perpetual shine upon him.

HENRY REED HOPKINS, M.D., (By request of the President.)
ELI H. LONG, M.D., Secretary.

---

AMONG the measures which failed of passage at the last session of Congress (*The Tribune*) was the bill intended to stop the use of phosphorus in the manufacture of matches. It failed despite the fact that it was supported by President Taft and that, apparently, there was no general sentiment in opposition to its object or to the method of accomplishing it by increasing the internal revenue tax on matches containing the obnoxious substance. An effort is to be made to secure the desired legislation at the special session.

The evils attending the use of phosphorus have been fully described. It often produces a gangrenous disease of the jawbone, called "phossy jaw," which disfigures the worker for life, and occasionally the mind is affected. There is no legitimate objection to stopping its employment, for there are harmless substances to take its place. The Diamond Match Company opened the way to the adoption of enlightened processes by offering to cancel a patent which covered the use of an inexpensive substitute. There ought to be no difficulty in securing proper action without long delay.

---

SIX men were arrested during the early part of April by the police and arraigned in the city court charged with expectorating on the sidewalk. Two were fined $2.00 each and the rest were released on suspended sentences, with reprimands and warnings.

---

GEORGE D. ELLIS, a commission merchant of Philadelphia, who was convicted last fall, of selling eggs declared to be unfit for food, surrendered himself to the Sheriff and was sent to the county prison to serve a sentence of three months, beginning April 1, 1911.

